# Supplementary material for: In-person training on COVID-19 case management and infection prevention and control: Evaluation of healthcare professionals in Bangladesh
Source: PLoS One. 2022 Oct 4;17(10):e0273809. doi: 10.1371/journal.pone.0273809 (PMC9531814; doi:10.1371/journal.pone.0273809)
Supplement: S1 Questionnaire — (PDF) [file pone.0273809.s001.pdf]

Knowledge Review Questions on IPC training for health care providers

1. The first case of novel coronavirus was identified in-

a) Beijing
b) Shanghai
c) Wuhan, Hubei
d) Tianjin
e) None of the above

2. SARS-CoV-1 (from the 2002-2003 outbreak) and SARS-CoV-2 (from today's outbreak) share the following trait:

a) They both bind to the hACE2 receptor in the respiratory tract using "spike proteins"
b) They are both parasitic infections
c) They both originated from Saudi Arabia
d) They have similar case-fatality rates
e) They both are spread by fecal-oral transmission

3. What is suspected to be the most common mode of transmission of COVID-19?
a) Airborne
b) Droplet.
c) Fecal-oral
d) Physical contact
e) All of the above

4. What is true of a potential COVID-19 vaccine?

a) SARS-CoV-2 is a good candidate microbe for vaccine development
b) One potential vaccine target is the Spike or S protein of SARS-CoV-2
c) Current strategies have been used for other infectious agents
d) Several strategies are currently being used to develop a COVID-19 vaccine
e) All of the above

5. You are evaluating the breathing of a patient. They are awake and speaking but you notice their SpO2 is 86%. What is the next step?

a) Move on to circulation and come back
b) Ignore it and move on
c) Place them on supplemental oxygen
d) Intubate them immediately
e) Wait for Chest X-ray

6. What specific feature of COVID infection allows it to spread silently from person to person?

a) Asymptomatic spreaders
b) Failure to use the right kind of facemask
c) More infectious than measles
d) We know very little about the virus
e) All of the above

7. The three steps of screening in the correct order are:

a) Identify, Immobilize, Inform
b) Identify, Isolate, Inform
c) Immobilize, Inform, Identify
d) Inform, Identify, Isolate
e) None of the above

8. The goal of triage is to:

a) Move the least sick patients back first as they are the fastest to treat.
b) Organize arriving patients in alphabetical order to maintain a clear record.
c) Identify patient's acuity level and prioritize care for the sickest.
d) All of the above
e) None of the above

9. Strategies to reduce potential spread of COVID-19 in the waiting room include:

a) Masks for symptomatic patients
b) Distancing of symptomatic patients at least 1 meter from others
c) Signs instructing patients how to protect others when they cough
d) Hand hygiene stations
e) All of the above

10. Social stigma related to health can lead people to:

a) Hide signs of illness to avoid discrimination
b) Seek out specialty medical care
c) More quickly adopt healthy behaviors
d) Discuss their illness on social media
e) None of the above

11. What are the recommended practices while we stay outside?

a) People should always follow physical distancing measures
b) Perform hand hygiene by washing hands frequently with soap and water or using alcohol-based hand rub
c) Follow good respiratory hygiene by covering your mouth and nose

d) Avoid touching your eyes, nose and mouth
e) All of the above

12. What areas should be prioritized for disinfection in non-health care settings?

a) Door and window handles,
b) Kitchen and food preparation areas,
c) Bathroom surfaces, toilets and taps,
d) Touchscreen personal devices, personal computer keyboards, and work surfaces
e) All of the above

13. Which of the following lower-cost solutions can be used for environmental disinfecting as an alternative to standard disinfectants?

a) 0.5% sodium hypochlorite solution (diluted bleach)
b) Alcohol-based hand gels
c) 0.01% sodium hypochlorite solution (diluted bleach)
d) Pure chlorine solution
e) All of the above

14. A patient with respiratory distress and a fever has been roomed into an isolation room in your Emergency Department. The patient has a known COVID-19 exposure. The patient needs to be intubated. What personal protective equipment should every staff member in the room be wearing during the intubation

a) Gloves, N95 mask, gown
b) Gloves, N95 mask, eye protection, gown, hair/shoe covers
c) Gloves, triple layer medical mask
d) Gloves, triple layer medical mask, eye protection, hair/shoe covers
e) Only surgical masks

15. Regarding supplemental oxygen, which is correct?

a) Nasal cannula is not appropriate to provide low flow Oxygen
b) Face mask can provide oxygen upto 5 L/min
c) Addition of 1 L oxygen will increase FiO_2 by 4%
d) Addition of 1 L oxygen will increase FiO_2 by 6%
e) In COVID-19, Outcome is better with mechanical ventilator than with high flow Oxygen

EVALUATION FOR SELF_ASSESSMENT:

1. Do you currently work with COVID-19 patients?

a) Yes
b) No
c) Prefer not to answer

2. Do you anticipate that you will work with COVID-19 patients in the future?

a) Yes
b) No
c) Prefer not to answer

3. How strongly do you agree with the following statement?

I have received adequate training on managing patients with COVID-19.

a) Strongly disagree
b) Somewhat disagree
c) Somewhat agree
d) Strongly agree
e) Prefer not to answer

4. How strongly do you agree with the following statement?

I have adequate access to information on managing patients with COVID-19.

a) Strongly disagree
b) Somewhat disagree
c) Somewhat agree
d) Strongly agree
e) Prefer not to answer

5. How strongly do you agree with the following statement?

I feel confident I can recognize clinical signs of COVID-19.

a) Strongly disagree
b) Somewhat disagree
c) Somewhat agree
d) Strongly agree
e) Prefer not to answer
