# Supplementary material for: In-person training on COVID-19 case management and infection prevention and control: Evaluation of healthcare professionals in Bangladesh
Source: PLoS One. 2022 Oct 4;17(10):e0273809. doi: 10.1371/journal.pone.0273809 (PMC9531814; doi:10.1371/journal.pone.0273809)
Supplement: S1 Table — (DOCX) [file pone.0273809.s002.docx]

**Supplementary Table 1: Change of knowledge between pre-test and post-test among participants**

| Pre-test | Frequency (%) | Post-test | Frequency (%) |
| --- | --- | --- | --- |
| Poor | 41 (11.48) | Fair | 1 (2.44) |
|  |  | Average | 27 (65.85) |
|  |  | Poor | 13 (31.71) |
| Average | 210 (58.82) | Fair | 60 (28.57) |
|  |  | Average | 136 (64.76) |
|  |  | Poor | 14 (6.67) |
| Fair | 106 (29.69) | Fair | 96 (90.57) |
|  |  | Average | 10 (9.43) |
|  |  | Poor | 0(0) |
