# Supplementary material for: In-person training on COVID-19 case management and infection prevention and control: Evaluation of healthcare professionals in Bangladesh
Source: PLoS One. 2022 Oct 4;17(10):e0273809. doi: 10.1371/journal.pone.0273809 (PMC9531814; doi:10.1371/journal.pone.0273809)
Supplement: Attachment — Submitted filename: Rebuttal Letter_22062022.doc [file pone.0273809.s004.doc]

**Cover letter**

Date: June 22, 2022

To: Dr Sanjai Kumar

Academic Editor, *PLoS ONE*

From: Dr Lubaba Shahrin

Corresponding author

Manuscript Ref. : PONE-D-22-01969

Subject: Response on the **minor comments** of the academic editor and the reviewers of *PLoS ONE* on manuscript Ref.:  PONE-D-22-01969 entitled “**In-person training in COVID-19 Case management and infection prevention and control: Evaluation of healthcare professionals’ knowledge in Bangladesh**”

Dear Dr Sanjai Kumar,

Thank you for providing us the opportunity to resubmit our manuscript following **minor revision**. We greatly appreciate the helpful comments of the reviewers and have attempted to address them in full. We now submit two drafts of the revised manuscript, one clean version and one which highlights the changes that we have made in red, as well as this rebuttal letter which provides a point-by-point response to the reviewers’ comments.

Kindly consider our revised draft for further in your prestigious journal. Please find the reviewers response as below.

**Reviewer’s comments:**

Reviewer #1: The manuscript “In-person training in COVID-19 Case management and infection prevention and control: Evaluation of healthcare professionals’ knowledge in Bangladesh” by Shahrin et. al., describes the importance of implementing the structured in-person training program for healthcare workers regarding case management in a pandemic.
Authors of this manuscript showed the effectiveness of in-person training program, where they compared participants’ pre and post training scores in epidemiology, clinical manifestation, management of case and infection prevention. Healthcare workers were clearly benefitted through this training program which was planned for better preparedness in controlling current or future disease outbreaks in Bangladesh.
I consider that the manuscript of Shahrin et. al., is clearly written and their claims are fully supported by well-designed study and proper statistical analysis. They obtained proper ethical approval for this study. Authors have responded to the issues found in earlier review. Authors recreated figure 1 & 2, which are now very clear to the readers. After revision with scientific editor the quality of the manuscript improved a lot.
So, I recommend the publication of this manuscript in PLOS ONE after minor correction.

**Comments:** Thank you very much for the encouraging comments

Minor comments: But still there are some minor typographical errors, that needs to be corrected in my opinion. Like in line 143, 184, 186, 197-198, 202, 208, 226 (The approval had ID-ACT-01112).

**Comments:** We have addressed the above-mentioned comments point by point and marked in underlined in the track changed version.

We hope and pray that the respected reviewer and academic editor will accept the revised drafts and allow us for the publication.
